# Schizophrenia and psychotic symptoms in families of two American Indian tribes

**DOI:** 10.1186/1471-244X-7-30

**Published:** 2007-06-28

**Authors:** Robert W Robin, Irving I Gottesman, Bernard Albaugh, David Goldman

**Affiliations:** 1 Director Hopi Guidance Center, P.O. Box 401, Second Mesa, AZ 86043, USA; 2 Departments of Psychiatry and Psychology, University of Minnesota Medical School, F235/2A West, 2450 Riverside Ave., Minneapolis, MN, 55454, USA; 3 Center for Human Behavior Studies, 10264 N 2422 CIR, Weatherford, OK, 73096, USA; 4 Laboratory of Neurogenetics, National Institute on Alcoholism and Alcohol Abuse, 5625 Fishers Lane, Room 3S-32, Rockville, MD, 20852, USA

## Abstract

**Background:**

The risk of schizophrenia is thought to be higher in population isolates that have recently been exposed to major and accelerated cultural change, accompanied by ensuing socio-environmental stressors/triggers, than in dominant, mainstream societies. We investigated the prevalence and phenomenology of schizophrenia in 329 females and 253 males of a Southwestern American Indian tribe, and in 194 females and 137 males of a Plains American Indian tribe. These tribal groups were evaluated as part of a broader program of gene-environment investigations of alcoholism and other psychiatric disorders.

**Methods:**

Semi-structured psychiatric interviews were conducted to allow diagnoses utilizing standardized psychiatric diagnostic criteria, and to limit cultural biases. Study participants were recruited from the community on the basis of membership in pedigrees, and not by convenience. After independent raters evaluated the interviews blindly, DSM-III-R diagnoses were assigned by a consensus of experts well-versed in the local cultures.

**Results:**

Five of the 582 Southwestern American Indian respondents (prevalence = 8.6 per 1000), and one of the 331 interviewed Plains American Indians (prevalence = 3.02 per 1000) had a lifetime diagnosis of schizophrenia. The lifetime prevalence rates of schizophrenia within these two distinct American Indian tribal groups is consistent with lifetime expectancy rates reported for the general United States population and most isolate and homogeneous populations for which prevalence rates of schizophrenia are available. While we were unable to factor in the potential modifying effect that mortality rates of schizophrenia-suffering tribal members may have had on the overall tribal rates, the incidence of schizophrenia among the living was well within the normative range.

**Conclusion:**

The occurrence of schizophrenia among members of these two tribal population groups is consistent with prevalence rates reported for population isolates and in the general population. Vulnerabilities to early onset alcohol and drug use disorders do not lend convincing support to a diathesis-stressor model with these stressors, commonly reported with these tribes. Nearly one-fifth of the respondents reported experiencing psychotic-like symptoms, reaffirming the need to examine sociocultural factors actively before making positive diagnoses of psychosis or schizophrenia.

## Background

Schizophrenia is a severe brain disease resulting in substantial loss of reproductive fitness in the past, but it is has a lifetime prevalence close to 1% worldwide and across cultures. It is clear that many individuals who are genetically vulnerable to schizophrenia do not express it, and that schizophrenia is multifactorial, involving genes, environmental factors, their interactions, and hypothesized epigenetic factors. The evidence includes monozygotic twin (MZ) concordances of about 50%, fraternal (DZ) rates about 15%, high rates of schizophrenia in the offspring of the normal twin within discordant MZ pairs, high rates in adoptees with a schizophrenic biological parent and normal adoptive parents, as well as the rapid decline of vulnerability to schizophrenia with decreasing genetic relationship to a schizophrenic proband [1].

Strategies to understand the variable expression of vulnerability to schizophrenia – the gene/environment interaction – include elucidation of the genetic risk factors, a focus on endophenotypes or intermediate phenotypes including premorbid cognitive differences shared by schizophrenics and their unaffected relatives, and identification of environmental triggers including prenatal, developmental and infectious agents. Cross-population studies can evaluate the potential effects of multiple differences between societies on prevalence or expression of schizophrenia including sociocultural differences. The risk of schizophrenia is thought to be higher in populations whose pre-existing cultural isolation has been recently disturbed [2,3], and schizophrenia may be precipitated by these socio-environmental stressors [4,5].

Isolated populations provide an excellent setting to identify both genetic and environmental etiologies of schizophrenia. The arguments for the use of isolates for genetic etiology are well known. There are likely to be fewer, or, increased susceptibility genes for a particular complex disease in genetic isolates as a result of the population having passed through a "bottleneck". Genetic linkage and linkage disequilibrium mapping methods are more readily applied because of reductions in disease gene and marker locus heterogeneity and better defined sampling frameworks. For example, only one or two major ancestral Y-haplotypes were found in South and Central American Indian populations ([6] and in Finns [7] and genetic diversity across a range of autosomally located short tandem repeat loci [STRs] was reduced about 15% across American Indian populations. These are findings indicative of reduction in genetic diversity.

Reductions in environmental heterogeneity in isolates and opportunities to observe effects of environmental factors- including cultural discontinuities – have sometimes been less well appreciated. We were able to investigate the prevalence and phenomenology of schizophrenia in a Southwestern Indian tribe and a Plains Indian tribe – which represent isolates in both the genetic and cultural senses. Both populations evidence little genetic admixture and virtually none to non-Indians. These populations were evaluated as part of a broader gene-environment investigation of alcoholism and other psychiatric disorders. Ascertainment was unbiased by the schizophrenia diagnosis or ascertainment for any disease. The diagnosis of main focus for the study had been alcoholism, and the prevalence of alcoholism as well as other social, demographic and genetic characteristics is very similar in these family-ascertained population cross-sections to the source tribal populations. Most critically for the purposes of this study, both tribes have undergone accelerated change within the past 200 years resulting in dramatic upsurges in problems that were previously unknown to them (e.g., alcoholism, other substance dependencies, unemployment) or previously had been very different in magnitude and expression (e.g., violence, trauma, within family abuse). The prevalence and phenomenology of schizophrenia in two distinct American Indian tribal populations is reported.

## Methods

### Study participants and sampling strategy

A total of 331 Plains (PL) and 582 Southwestern (SW) American Indian adult participants were interviewed on the basis of family membership. Tribal elders provided information on multigenerational pedigrees. Three pedigrees for each tribe were selected on the basis of availability of adult members, geographic accessibility, and willingness of at least one family member to be an advocate for the study. The family pedigrees coalesce, so it is more accurate to regard each study population as a sub-sample of the overall tribal pedigree.

Participants gave informed consent under a human research protocol approved by the Institutional Review Board of the National Institute on Alcoholism and Alcohol Abuse, National Institutes of Health, and the respective tribal councils, and was conducted in compliance with the Helsinki Declaration. Focus groups comprised of tribal staff and community members reviewed testing instruments and procedures for participant recruitment and data collection. Community support was critical in attaining a high level of study participation. Employing members of the community for recruitment and portions of the data collection facilitated rapport with participants. Completion rates were noteworthy, yielding 98% of the PL and 94% of the SW eligible persons contacted.

Participants were 21 years of age or older and eligible for official enrollment in the SW and PL tribes (requiring ≥ 1/4th tribal blood). These populations were chosen for genetic transmission and linkage studies on alcoholism and psychiatric disorders. Due to the high prevalence of alcoholism in this population, there was no need to ascertain through an alcoholic proband. Pedigrees were selected on the basis of structure and accessibility only and without knowledge or reference to their clinical histories or to the clinical histories of relatives. Thus, there was no initial diagnostic bias. Also, pedigrees were exhaustively analyzed and data were obtained on nearly all members of the families including those on and off-reservation. Participants represented approximately 10% of the adult members of the SW tribe and 12% of the adult members of the PL tribe.

We used genealogical information to address the extent to which the family-derived samples were genetically representative. For the SW tribe, the fraction of genes shared by common descent was calculated for 169,071 pairs of participants. A randomly drawn pair of individuals shared 1.2% of their genes by descent, indicating that most pairs have a low degree of relationship. The average degree of relationship for the whole tribe has not been measured but the average sharing by descent of 1.2% is compatible with the tribal population's size and history. Preliminary analyses for the PL tribe by Bernard Albaugh in a manuscript under preparation indicate a similar average degree of genetic relationship.

Detailed genetic, demographic, and social data allowed comparison of the representativeness of the samples to the overall populations of the two tribes as could be evaluated using US Census data. The data indicate representative samples, except for some definitional variations such as incorporating common-law relationships of > 1 year into the "marriage" category [8].

Comparative rates of schizophrenia in this study's two American Indian tribal groups, in other isolate populations, and in homogeneous populations were analyzed using 95% Confidence Intervals and Standard Error measures.

### Interviews and psychiatric diagnoses

Interviews were conducted with a modified version of the Schedule for Affective Disorders and Schizophrenia-Lifetime version (SADS-L) [9]. Probes were added to allow diagnoses utilizing both Research Diagnostic [10] and Diagnostic and Statistical Manual of Mental Disorders, Third Edition-Revised (DSM-III-R) criteria [11], and to limit cultural biases. The SADS-L has well-established validity in population and clinical studies [12] and is reliable when administered to American Indians by clinicians experienced with psychiatric assessment in these populations [13-16]. Diagnoses were sometimes augmented by collateral information from educational, court, and medical records and in collaboration with family members. In several cases, additional information was obtained when participants showed evidence of a current psychotic episode or where memory impairment was evident.

A psychologist and a clinical social worker with a combined total of 40 years experience in psychiatric assessment in diverse American Indian populations administered the SADS-L interviews. Clinical interviews were rated blindly for DSM-III-R lifetime and point prevalence diagnoses yielding good to excellent rates of agreement between two independent blind raters (kappa coefficients ranged from 0.63 to 1.00). Diagnostic differences were resolved in a consensus conference that included a senior psychiatrist experienced in the psychiatric assessment of American Indians. Provisions were made to limit diagnostic errors due to culture-specific phenomena. For example, visual and auditory hallucinations that occurred within the context of American Indian ceremonies and rituals were not considered to be indicative of psychosis or schizophrenia [17,18].

Frequency of response to six symptoms for psychosis was calculated for each tribe. The six symptoms were: (1) heard voices that could not be heard by other people, (2) had visions or saw things that were not visible to other people, (3) had strange bodily feelings, (4) had beliefs later found out not to be true, (5) did something to call attention to oneself, and (6) people had difficulty understanding what subject was saying because of mixed up speech and lack of sense. Positive responses that were attributed to withdrawal symptoms or alcohol psychosis were coded in the negative. Probes and additional questions (e.g., delusions, formal thought disorder, duration, number of episodes) were added. Due to incomplete responses, four (4) SW and 69 PL individuals were completely eliminated from the analyses. Another 18 individuals did not respond to at least one of the six schizophrenic symptoms, further reducing the N for analysis of the individual symptom items.

## Results

We investigated the prevalence and phenomenology of DSM-III-R schizophrenia in 329 females and 253 males from a SW tribe, and in 194 females and 137 males of a PL tribe [Table 1]. As described in Methods, the two tribal samples were representative in terms of degree of relationship and demographic features, and, as described elsewhere, they were similar in distribution for gender, age, and education (Robin et al., 1999).

**Table 1:** Comparative rates of schizophrenia in isolate and homogenous populations

	Southwest Indians	Plains Indians	Roy, et al^1 ^[56]	Sampath, et al^2 ^[57]	Kinzie, et al^3 ^[58]	Kebede, et al^4 ^[59]	Myles-Worsley, et al^5 ^[60]	Scully, et al^6 ^[62]	McConnell, et al^7 ^[61]	Bates, Van Dam^8 ^[64]	National Comorbidity Study^9 ^[63]
N	582	331	1218	214	131	68,378	13,750	29,542	1242	12,200	8098
Prevalence per 1000	8.6	3.02	22.17	29.58	21.00	4.69	11.64	3.89	2.42	3.1	5.00
95% C.I.	± 2.4	± 1.1	± 2.6	± 2.8	± 2.5	± 1.2	± 2.0	± 1.2	± 0.9	± 1.2	± 1.4
S.E.	0.012	0.006	0.013	0.014	0.013	0.006	0.01	0.006	0.005	0.0005	0.007

^1^1970, Saskatchewan Reservation, age 15+; ^2^1974, Baffin Island Eskimo, age 15+; ^3^1988, Western United States Native Americans, age 20+; ^4^2003, Butajira-Ethiopia, age 15-49; ^5^1999, Palau, Micronesia, age 15+; ^6^2004, Co. Monaghan, Ireland, age 0+; ^7^2002, District of Derry, Ireland, age 18-64; ^8^1984, British Columbia Coastal Indians; ^9 ^1994, United States population-based, age 15-54

Of the 582 SW Indian respondents, five (4 M, 1 F) were diagnosed with schizophrenia (Prevalence = 8.6 per 1000). Prevalence was 3.02 per 1000 in the PL Indians (1/331, 1 M). Two SW Indians were in a current episode at the time of interview. Age of onset for schizophrenia was 14,15, 17,19, and 26 for the five SW Indians, and age of onset was 22 for the one PL Indian. All five SW schizophrenics reported psychotic symptoms, and four of them had four or more positive responses out of six possible. The PL schizophrenic had five positive responses.

Each SW Indian schizophrenic had at least two other lifetime psychiatric disorders. These included alcohol dependence or abuse (5/5), drug use disorder (5/5), and antisocial personality disorder (3/5). The abused drugs were cannabis (4/5) and inhalants (4/5). One schizophrenic reported hallucinogen abuse. In every case, onset of schizophrenia was preceded by either alcohol (mean age = 14) or drug abuse (mean age = 11). The PL schizophrenic had agoraphobia but no indication of a substance abuse disorder.

Many of the non-schizophrenic SW (N = 573) and PL (N = 261) Indians also had one or more symptoms of schizophrenia. 21% (120/573) of the non-schizophrenic SW Indians responded positively to at least one of the initial six standardized questions for psychosis, and 12% (67/573) responded positively to two or more questions. Auditory and visual hallucinations were reported by 15% (86/573) of the non-schizophrenic participants and reported together 84% (72/86) of the time. The other four symptoms (strange feelings in body, false beliefs or ideas, calling attention to oneself with strange behavior, speech difficult to understand) were reported by 2% to 5% of SW Indians.

Approximately 17% (44/261) of the non-schizophrenic PL Indians had at least one psychotic symptom, and 10% (27/261) responded positively to two or more symptoms. Auditory and visual hallucinations were reported by 10% of the participants and co-occurred 93% of the time. The other four psychotic symptoms were reported by 1% to 4% of PL respondents.

None of the five affected SW Indians were first-degree relatives. However, there were some common elements in their social backgrounds. Four of the five SW schizophrenics had been removed from their homes as children and placed into foster care or missionary school as compared to 13% of the non-schizophrenics. Three of the four questioned about childhood sexual abuse reported being sexually abused prior to the age of sixteen. Four had been arrested and jailed. Their educational levels varied. Two dropped out of school after the 5^th ^and 9^th ^grades, and the other three graduated from high school. The PL Indian with schizophrenia had no reported history of childhood problems and completed high school.

## Discussion

Worldwide studies on the prevalence of schizophrenia [Table 1] suggest considerable variation [19,20]. As can be seen, the studies include homogenous and isolate populations from Micronesia, Ethiopia, Ireland, arctic Canada, and American Indian tribal groups, as well as cosmopolitan populations. Comparative rates of schizophrenia in these populations range from 2.4 per 1000 [21] to 29.58 per 1000 [22].

Data on the prevalence of schizophrenia in American Indians is mixed. A lifetime prevalence rate of schizophrenia of 22.7 per 1000 was identified among Indians from Saskatchewan, contrasting with a rate of 2.4 per 1000 for non-Indians living in the same region [22], but not really different from rates of about 1-3% found in other populations worldwide. The lifetime rate of schizophrenia was 3% for a Northwestern Indian tribe [23] and 8% for a Baffin Eskimo settlement [24].

In all of these studies, sample sizes were small or modest, and with only a few exceptions, no standardized psychiatric interviews with well-defined criteria for schizophrenia were used. In previous studies of North American Indians, it's likely that the prevalence of schizophrenia was "overestimated in early clinical and epidemiological studies because of the tendency for North American psychiatrists to misdiagnose bipolar affective disorder as schizophrenia" [25]. A failure to use standardized instruments and criteria can result in a misdiagnosis of an individual's disturbed or psychotic-like behavior whether drug-induced, catalyzed by depression, or indicative of another psychiatric condition [26].

A common problem in cross-population studies is diagnostic variation [27]. Reported findings of schizophrenia among American Indians have also been hampered by their reliance on small clinical samples and a failure to adequately consider the role of substance abuse in lending expression to psychotic symptoms [28-31]. A further limitation concerns the accuracy and validity of diagnoses for schizophrenia and other psychotic disorders, often thought to have been made with little or no awareness of culturally bound syndromes specific to American Indian societies [32]. Nevertheless, only Sampath's 1974 study demonstrated substantial lifetime prevalence rates above the norm.

The occurrence of schizophrenia within these two distinct American Indian tribal groups is consistent with the 0.3-2.8% [33] and 1.3% [34] life-time expectancy rates reported for the general United States population and below the approximate 2.5% risk reported for second-degree relatives [4,35]. While we were unable to factor in the potential modifying effect that mortality rates of schizophrenic tribal members may have had on the overall tribal rates, the incidence of schizophrenia among the living was well within the normative range.

The fact that schizophrenia prevalence rates for these two American Indian populations groups do not appear to be different from most other population groups is striking considering the degree by which they have undergone cultural change and are rife with stress. These stressors have been exacerbated by the SW and PL tribal groups' exposure to accelerated and dramatic lifestyle changes resulting in poverty and low socioeconomic conditions [36], out-of-home placements [37], and for the SW tribal group, frequent exposure to lifetime traumatic events including child sexual abuse [38,39]. Nevertheless, these tribal groups' rates of schizophrenia remain consistent with those found in most other populations despite the presence of significant stressors hypothesized – within the context of the diathesis-stressor model – to catalyze the expression of schizophrenia.

None of the diagnosed schizophrenic study participants were closely related to each other, but as might be expected from the inheritance of schizophrenia, were more related than average. On the other hand, no one genetic cluster is represented, none are siblings, and two of the five are unrelated to any of the others [Table 2].

**Table 2:** Kinship of schizophrenic and non-schizophrenic Southwestern Indians. Kinship of five schizophrenic and 576* non-schizophrenic Southwestern Indians

Schizophrenic Individuals	A	B	C	D	E	Non-schizophrenic Controls (N = 576) Mean (S.D.)
#2296 A	-	0.249	0	0	0.120	0.011 (0.039)
#2302 B		-	0	0	0.126	0.011 (0.039)
#3410 C			-	0	0	0.016 (0.068)
#3448 D				-	0	0.003 (0.031)
#3976 E					-	0.013 (0.053)

Mean kinship among the ten pairs of schizophrenics = 0.0495. Mean kinship relationship of schizophrenics to all others = 0.0111.

* Among the 577 non-schizophrenic study participants, one could not be located in the pedigree file. Therefore, this analysis pertains to 576 participants.

Interviews with their first-degree relatives revealed no previous history of family schizophrenia, though our study sample was limited in that not all family members were interviewed and histories of schizophrenia did not extend beyond three generations. Nevertheless, extensive super pedigrees were sampled with the study findings lending support to the likely etiologic heterogeneity of schizophrenia in these two tribal populations [40]. However, the fact that many of the study participants were part of "superfamilies" can cause some noise in the calculation of prevalences.

Specific vulnerabilities lending support to a diathesis-stressor model were in evidence, however, life stressors are common among American Indian societies. Most striking is that neither tribe had a high prevalence of schizophrenia despite reported stressful socioeconomic conditions and high prevalence rates of alcohol and drug use disorders. Substance abuse disorders preceded the onset of schizophrenia in all but one of the six diagnosed schizophrenics, a common finding in studies of inpatient schizophrenic populations [41-43]. Cannabis abuse in particular has been found in clinical and epidemiological studies to exacerbate the symptoms of schizophrenia with indications that it may precipitate schizophrenia [44,45]. Similar findings have been reported for inhalant abuse when comparing schizophrenic groups, both with other psychiatric patient groups and normal subjects [46-49]. Yet despite high rates of alcohol and other drug use disorders in the two American Indian tribal groups under study, the prevalence rates of schizophrenia were not elevated. Other commonly identified stressors included child sexual abuse and out-of-home placement. Exposure of populations with psychosis and schizophrenia to child physical and sexual abuse has seldom been investigated, but the few inpatient studies available report significant associations between these conditions [50-52].

Nearly 20% of a combined 840 tribal study participants responded positively to questions about psychotic symptoms that were not related to the use of substances such as alcohol. This percentage is similar to findings of delusions, paranoid thoughts, and other psychotic symptoms reported to occur in the general population [53-55], though the underlying manifestations for the expression of psychotic symptoms may differ for American Indian people. Reported symptoms for the two American Indian groups under study were most often described within a traditional cultural and spiritual context. For example, in these tribal groups a common symptom of bereavement is seeing or hearing the deceased person at which time prescribed ways are invoked to provide relief to the bereaved. Another commonly reported experience is participation in traditional ceremonies such as the Sundance and sweat lodges. These events intentionally alter the participant's physical, emotional, and spiritual status in which visions and paranormal experiences are expected to occur. Vision Quests are another activity that can alter one's consciousness. In this instance, the individual travels to a remote sacred location, abstains from food and water for several days with limited or no personal contact. The expectation in these circumstances is to gain perspective and knowledge by experiencing visions.

The occurrence of psychotic symptoms in non-schizophrenics demonstrates that the diagnosis for schizophrenia is not to be made by finding evidence of a few psychotic symptoms, but by meeting a sufficient group of finite criteria. In the case of these two American Indian tribal groups, the phenomenology of schizophrenia appears classic – the non-familiality is consistent with the multifactorial-polygenic nature of schizophrenia and the comorbid diagnoses are consistent as well. While it remains an open-ended question whether schizophrenia is more common in certain populations, shedding evidence on etiology is the best evidence. Our findings demonstrate that when schizophrenia is carefully measured, it has been found to be neither especially abundant nor rare in American Indian tribal groups despite the fact they have factors that have been asserted to be etiologic or triggering.

## Conclusion

In sum, it appears that the occurrence of schizophrenia among members of these two tribal population groups is consistent with prevalence rates reported for most population isolates and in the general population. While the number of diagnosed schizophrenics was too small to subject to refined statistical analysis, we found similarly reported associations with substance abuse and childhood deprivation and abuse. Finally, nearly one-fifth of the respondents reported experiencing psychotic-like symptoms. At least in these two tribal groups, the number of positive responses to what are intended to be indices of psychotic behavior reaffirms the need to consider sociocultural factors actively before making positive diagnoses of psychosis or schizophrenia.

## Competing interests

The author(s) declare that they have no competing interests.

## Authors' contributions

RR performed psychiatric interviews, organized and provided oversight for blind ratings and adherence to standardized diagnostic criteria, was responsible for field operations for the SW American Indian tribal group including study participant recruitment and construction of genealogies, designed and selected assessment measures and testing instruments, and drafted the first version of the manuscript. IIG performed statistical analysis and contributed to the writing of subsequent drafts. BA performed psychiatric interviews, was responsible for field operations for the Plains American Indian tribal group including study participant recruitment and construction of genealogies, and contributed to the manuscript. DG conceived the study, participated in its design and implementation, and contributed to the writing of the manuscript. All authors read, gave feedback, and approved the final manuscript.

## Pre-publication history

The pre-publication history for this paper can be accessed here:


